# The relationship between non-suicidal self-injury and childhood abuse in transgender people: a cross-sectional cohort study

**DOI:** 10.3389/fpsyg.2023.1062601

**Published:** 2023-08-09

**Authors:** Qiang Cao, Qi Zhang, Yuquan Chen, Zixu He, Zhibiao Xiang, Haoran Guan, Na Yan, Yi Qiang, Mantao Li

**Affiliations:** ^1^Department of Earth Sciences, Kunming University of Science and Technology, Kunming, China; ^2^School of Medicine, Macau University of Science and Technology, Macau, Macao SAR, China; ^3^Undergraduate Department, Taishan University, Taian, China; ^4^Chinese Academy of Medical Sciences, Beijing, China; ^5^Undergraduate Department, University of New South Wales, Sydney, NSW, Australia; ^6^Xiangya School of Medicine Central South University, Changsha, China

**Keywords:** transgender, childhood abuse, non-suicidal self-inflicted injury, intermediary effect, mental health

## Abstract

**Objective:**

To explore the relationship between non-suicidal self-injury (NSSI) and childhood abuse in transgender people and the mediating effect of emotional dysregulation traits in the association between childhood abuse and non-suicidal self-injury.

**Patients and methods:**

From May to October 2021, 296 female-to-male (FTM) and 675 male-to-females (MTF), with age of 24.5 ± 6.4 years, were recruited using peer-driven sampling and anonymous questionnaires in Guangdong Province. The Childhood Abuse Questionnaire (CTQ-SF), the Personality Diagnostic Questionnaire (PDQ-4+) emotion regulation ability scale and the DSM-5 Clinical Examination of Stereotypic Disorders were used to measure childhood abuse experiences, emotional dysregulation traits and self-injurious behaviour, respectively.

**Results:**

Childhood abuse scores were positively correlated with both emotional dysregulation traits scores and non-suicidal self-injury (NSSI) behaviours (*p* < 0.01), and emotional dysregulation traits scores were positively correlated with NSSI behaviours (*p* < 0.01); emotional dysregulation traits partially mediated the association between childhood abuse and NSSI behaviours, with the mediating effect accounting for 23.23% of the total effect. In addition, among the factors of childhood abuse, emotional dysregulation traits mediated the association between emotional abuse, emotional neglect, sexual abuse, physical abuse, physical neglect and NSSI behaviour significantly, with the mediating effect accounting for 22.48%–32.58% of the total effect.

**Conclusion:**

Transgender NSSI behaviours are associated with childhood abuse and emotional dysregulation traits, and emotional dysregulation traits partially mediates the association between childhood abuse and NSSI behaviours, and screening for emotional dysregulation traits in transgender people and timely interventions are needed to improve the current situation of discrimination against transgender people.

## Introduction

Non-suicidal self-injury (NSSI) is defined as direct, intentional damage to one’s own bodily tissues that is not intended to be suicidal and is not socially acceptable or sanctioned (Peng et al., 2019; Liu et al., 2020). Although most NSSI behaviour does not directly result in death, it is associated with the onset of clinical emergencies, functional impairment, greatly increases the risk of suicide attempts and is a risk factor for death by suicide (Suen et al., 2021; She et al., 2022). Owing to the persistent rise in social perceptions and family stress, transgender individuals are more prone to weakened psychological defenses, poor self-control, and susceptibility to peer influence compared to cisgender individuals (Tang et al., 2016; Chen et al., 2019). As a result, transgender people are more likely to engage in NSSI behaviour. In the context of traditional Chinese society, transgender individuals face heightened pressure due to rigid societal norms and expectations. The influence of Confucian principles, which emphasize familial obligations and societal harmony, often translates into a potent cultural bias towards heteronormativity and strict binary gender roles. Transgender individuals who deviate from these traditional expectations can be subjected to intense scrutiny and marginalization. Furthermore, filial piety, a core value in Chinese society, often places an added burden on transgender individuals, who may face estrangement from their families upon disclosing their identities. Consequently, the traditional Chinese societal backdrop adds multiple layers of stress and discrimination for transgender people, beyond the usual hardships encountered by this community globally. This context underscores the need for robust, culturally-sensitive support systems and societal reforms to protect and affirm transgender individuals within the Chinese society.

Childhood abuse as an influential factor in the occurrence of NSSI behaviour has attracted the attention of scholars nationally and internationally (Duan et al., 2021). Psychiatric Department, University Town Hospital Affiliated to Chongqing Medical University, Chongqing has done a survey, study of transgender people showed that approximately 47% of transgender people had experienced NSSI behaviour in their lifetime, with approximately 68% experiencing at least one type of childhood abuse and approximately 55% experiencing multiple types of childhood abuse (Fan et al., 2021). For individuals, childhood abuse not only has a short-term impact on their NSSI behaviour in adolescence, NSSI risk may carry over into adulthood. However, NSSI behaviour is more common in adolescents (Wang et al., 2021). Borderline Personality Disorder (BPD) is a common and problematic clinical problem that manifests as emotional instability, impulsive behaviour, interpersonal tension and suicidality, and BPD is often co-occurring with NSSI behaviour. The emotional dysregulation traits are a core characteristic of BPD. Although emotional dysregulation traits are a core characteristic of Borderline Personality Disorder, it is important to note that assessment of these traits alone cannot fully determine the development of BPD in an individual. However, assessing emotional dysregulation traits can provide insight into the risk of developing BPD in a group (Kröger et al., 2011). Best et al.’s (2015) study have shown that BPD is present in up to 41.56% of patients with a history of NSSI behaviour or who are admitted to the hospital for self-injury. Sun et al.’s (2022) study shown that the prevalence of BPD in the NSSI group was found to be 40.9% in the NSSI group and 11.5% in the control group. Many scholars have explored the mechanisms of BPD, of which childhood abuse has a significant influence on the development of BPD (Yang et al., 2016; Yan et al., 2019). As personality traits are not fully stabilised in adolescence, personality is largely mature and more stable in adulthood (Zhang et al., 2016). Compared to the general population, transgender people, as a special group, are often not accepted by the general public, have greater mental health challenges and are more likely to commit suicide (Chang et al., 2019). The exploration of the causes and prevention of suicide has become the focus of research on the psychological problems of transgender people, and as NSSI is an important factor in suicide risk, the mechanisms that lead transgender people to engage in NSSI behaviour should be further investigated (Chen et al., 2020). In our previous study, we found that the reliability of the Borderline Symptom List-23 (BSL-23) questionnaire was low in the transgender population. The reason for the low reliability of the BSL-23 questionnaire may be that the original questionnaire was developed for the mentally ill population, but was not considered for use in the transgender population. Therefore, the Difficulties in emotion regulation ability scale (DERS), which has high reliability in the transgender population, was used to assess the high relevance to BPD in the transgender population by assessing emotional dysregulation traits in the transgender population (Bohus et al., 2009). The objective of this study is to investigate the relationship between NSSI behaviour, childhood abuse and emotional dysregulation traits in transgender people, and to explore the role of emotional dysregulation traits in the relationship between childhood abuse and NSSI behaviour, filling a gap in this area of research.

## Patients and methods

### Patients

In this study, MTF and FTM participants were recruited in Guangdong Province from May to October 2021 using a peer-driven sampling method, and an anonymous questionnaire survey was conducted. The peer-driven sampling method is a chain-referral sampling technique in which an initial set of participants, or “seeds, “recruits their peers, who in turn recruit their own peers, and so on. This approach can potentially reach hidden populations and improve the representativeness of the sample. SPSS 17.0 statistical analysis software is used and 1,209 of questionnaires were distributed which total of 1,021 questionnaires were returned, with 971 valid questionnaires, representing a responses rate of 95.10%. The study was completed in accordance with the Declaration of Helsinki as revised in 2013, informed consent was obtained from the subjects and approval was obtained from the Ethics Committee of Kunming University of Technology.

## Survey tools

### General information questionnaire

The main areas covered in the General Information Questionnaire, including gender, age, education level, only child or not, smoking, drinking, and the parents’ education level, were all provided in Mandarin. The survey was administered in Mandarin since it is the primary language of the study population. This was done to ensure that participants understood the questions clearly and could respond accurately.

### Childhood abuse questionnaire (CAQ)

Spielmann et al. (2022) Revised Childhood Abuse Questionnaire was used. The questionnaire consists of five dimensions, emotional abuse, emotional neglect, sexual abuse, physical abuse and physical neglect, with entries rated on a 5-point scale from 1 (never) to 5 (always) and some entries on a reverse scale, with a total score between 15 and 75. Childhood abuse was measured using the Revised Childhood Abuse Questionnaire which consists of five dimensions: emotional abuse, emotional neglect, sexual abuse, physical abuse, and physical neglect. Each dimension score was added to obtain the total childhood abuse score, which ranged from 15 to 75. This questionnaire was used with a translated version of Mandarin. Zhang’s (2011) study confirmed that the Mandarin version of the CAQ questionnaire has good reliability and validity and is suitable for use in various populations in China. The Cronbach’s alpha coefficient for this questionnaire in this study was 0.904.

### NSSI behavioural screening

The question on NSSI from the DSM-5 Definitive Clinical Examination of Disorders (SCID-5-CV) overview module was selected, i.e., “At any time in your life, have you ever attempted self-injury (self-harm that is not suicidal)?” If the answer to this question was “yes,” the NSSI group was considered.

### Difficulties in emotion regulation ability scale (DERS)

The DERS scale is considered applicable to all group types (including the transgender community). In this study, we used the Mandarin version of the DERS form compiled by Wang et al. This form has good reliability for Chinese groups and is applicable to this study (Wangguo et al., 2021). Therefore, the Mandarin version DERS scale was utilized to assess emotional dysregulation traits scores in the study participants, with items scored on a scale of 0 to 1, with “no” scored as 0 and “yes” scored as 1, and a threshold score of 9 The total score ranges from 0 to 17 (Peng, 2021). The Cronbach alpha coefficient for this questionnaire in this study was 0.949.

## Statistical analysis

SPSS 17.0 statistical analysis software was used for data processing in this study. Differences between groups in childhood abuse scores on demographic factors were analysed by *t*-test or one-way ANOVA, and differences between groups in emotional dysregulation traits scores on demographic factors were analysed by Wilcoxon rank test and Kruskal-Wallis H-rank test.

The utilization of both Nonparametric and parametric tests aims to form the fundamental basis of statistical inference. Parametric testing is a method of inferring the parameters of the overall distribution, such as mean and variance, when the overall distribution form is known. Nonparametric tests were used to compare differences in childhood abuse and emotional dysregulation trait scores across different demographic groups. The Z scores represent the significance of the difference between groups based on nonparametric tests. They are used to compare scores across different demographic groups in cases where the data are not normally distributed.

**Figure 1:**
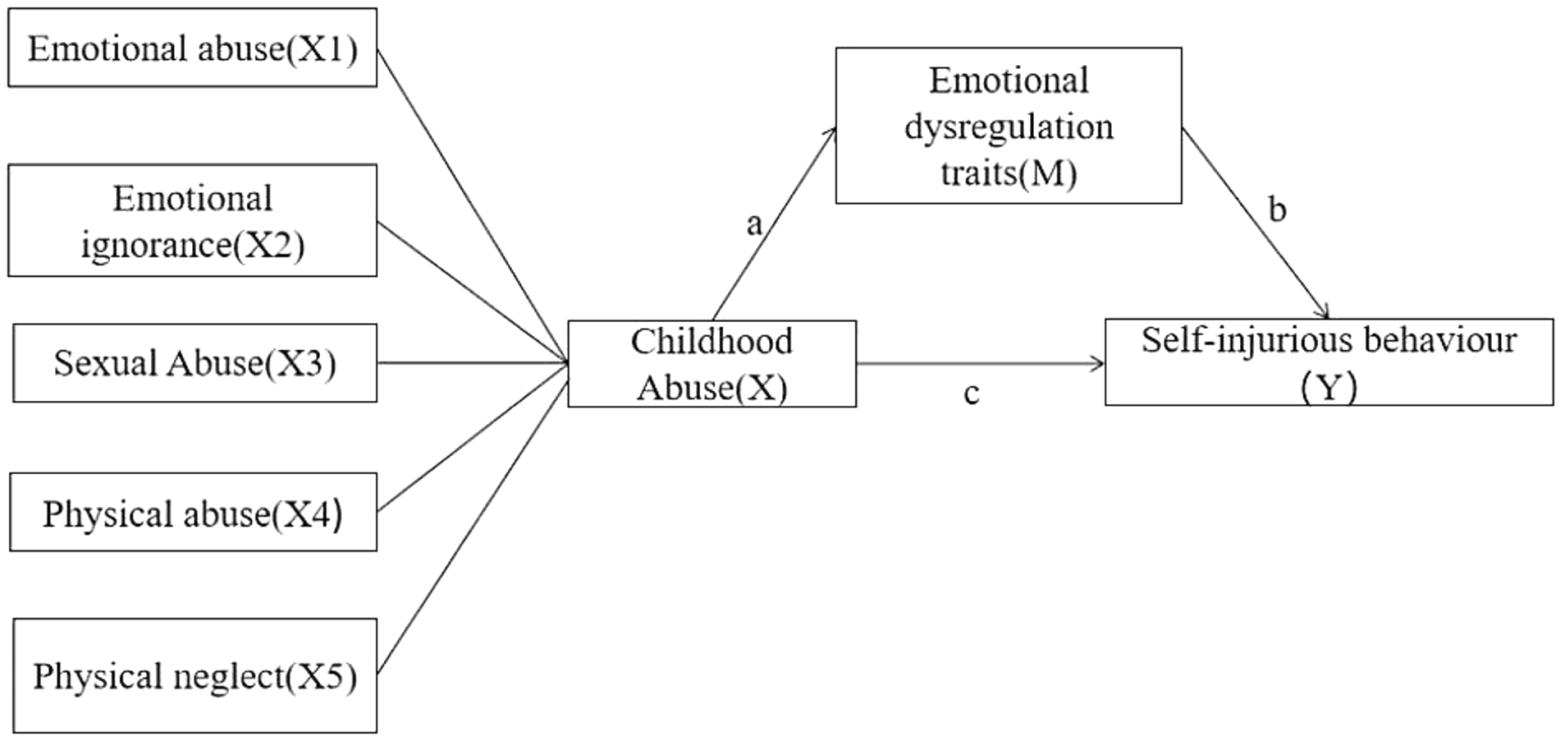
Schematic diagram of the intermediary effect analysis.

Pearson correlations were used to analyse the correlations between childhood abuse and its factors, emotional dysregulation traits and NSSI behaviour, with *p* < 0.05 being considered statistically significant. The mediation model in this study is shown in Figure 1, this study examined each of the five different types of abuse using this model to determine the relationship between childhood abuse, self-injurious behaviour and emotional dysregulation traits. This study used the bootstrapping method to develop a mediation model as a way to examine the mediating role of emotional dysregulation traits between childhood abuse, its factors and NSSI behaviour. To test for the mediating role of emotional dysregulation traits in the relationship between childhood abuse and NSSI behaviour, we used the PROCESS macro (model 4) for SPSS (version 17.0). The bootstrapping method was used to estimate the indirect effect, with 10,000 bootstrap samples drawn to compute bias-corrected confidence intervals.

## Results

### Common method deviation test

The data obtained were tested for the presence of common method bias using the Harmon one-factor test. The results of the analysis of all variables showed that 12 factors with eigenvalues greater than 1 were analysed, and the variance explained by the first factor was 3.341%, which was less than the critical threshold of 20%, so there was no serious common method bias and the data met the requirements of the mediation effect analysis.

### Demographic correlates of childhood abuse and emotional dysregulation traits among transgender

Of the 971 valid survey respondents, 296 transgender males and 675 transgender females, the age range of the respondents was 24.57 ± 6.42 years. 607 had NSSI behaviour, a detection rate of 62.51%, including 102 FTM and 505 MTF. Demographic differences were tested for NSSI behaviour, childhood abuse and emotional dysregulation traits. The results of the tests are shown in Table 1, with a mean score of 33.19 ± 6.97 for childhood abuse, the Childhood Abuse score of MTF (34.53 ± 6.907) was higher than that of FTM (29.41 ± 5.019) and 0.86 for emotional dysregulation traits. There were also significant differences in childhood abuse in terms of gender, educational level, marital status, whether or not the father smoked, and father’s education level, while there were significant differences in emotional dysregulation traits in terms of gender, whether or not the child was an only child, educational level, whether or not the mother drank alcohol, and mother’s education level.

**Table 1:** Demographic differences in scores on the questionnaire.

Variables	Number of people	Childhood abuse	*t*/*F*	*p*	Emotional dysregulation traits ( $\bar{X}$ )	*Z*	*p*
Sex			3.342	<0.001		2.322	0.002
MTF	675	34.53 ± 6.907			0.74		
FTM	296	29.41 ± 5.019			1.15		
Only child or not			0.125	0.421		2.091	0.014
Yes	537	33.19 ± 6.727			0.98		
No	434	31.84 ± 7.278			0.76		
Education level			8.723	<0.001		7.142	0.022
Lower secondary and below	129	39.02 ± 11.428			1.93		
High school	442	34.21 ± 7.941			0.76		
Tertiary education	247	32.66 ± 6.792			0.86		
Bachelor’s degree and above	153	29.84 ± 5.873			1.09		
Smoking			3.129	<0.001		0.875	0.161
Yes	391	31.95 ± 6.958			0.87		
No	580	30.21 ± 5.731			0.81		
Drinking			1.449	0.025		2.231	<0.001
Yes	104	33.07 ± 8.019			1.26		
No	867	32.86 ± 7.295			0.77		
Father’s education level			3.997	<0.001		4.518	0.004
Lower secondary and below	324	33.57 ± 8.241			0.94		
High school	256	31.25 ± 7.994			0.72		
Tertiary education	240	28.63 ± 5.904			0.63		
Bachelor’s degree and above	151	31.02 ± 6.073			0.99		
Mother’s education level			1.848	<0.001		6.043	0.007
Lower secondary and below	416	31.98 ± 6.366			0.94		
High school	344	30.51 ± 6.841			0.85		
Tertiary education	129	31.02 ± 5.984			0.87		
Bachelor’s degree and above	82	34.01 ± 9.147			0.91		
Next of kin with or without mental illness			−0.994	0.216		1.064	0.042
Yes	62	31.24 ± 8.142			0.92		
No	909	38.91 ± 10.943			1.94		

### Analysis of the correlation between the total childhood abuse score and its factor scores, emotional dysregulation traits score and NSSI behaviour

Correlation analysis revealed that Childhood Abuse, Emotional abuse, Emotional neglect, Sexual Abuse, Physical abuse, Physical neglect, Emotional dysregulation traits and Self-injurious behaviour were all strongly correlated with each other and could be further analysed. As shown in Table 2, all items were significantly correlated (*p* < 0.01). The correlation analysis was further conducted separately for the FTM and MTF groups. The correlations between Childhood Abuse, Emotional abuse, Emotional neglect, Sexual Abuse, Physical abuse, Physical neglect, Emotional dysregulation traits, and Self-injurious behaviour were significant for both genders, showing a strong relationship within each group.

**Table 2:** Correlations between childhood abuse, emotional dysregulation traits scores and NSSI behaviour (r).

Variables	SD	Childhood abuse	Emotional abuse	Emotional neglect	Sexual abuse	Physical abuse	Physical neglect	Emotional dysregulation traits	Self-injurious behaviour
Childhood abuse	3.02	1.000							
Emotional abuse	4.19	0.531^*^	1.000						
Emotional neglect	2.18	0.791^*^	0.236^*^	1.000					
Sexual abuse	3.63	0.152^*^	0.148^*^	0.158^*^	1.000				
Physical abuse	2.82	0.392^*^	0.452^*^	0.132^*^	0.119^*^	1.000			
Physical neglect	3.75	0.801^*^	0.213^*^	0.394^*^	0.117^*^	0.130^*^	1.000		
Emotional dysregulation traits	5.19	0.225^*^	0.358^*^	0.242^*^	0.186^*^	0.172^*^	0.115^*^	1.000	
Self-injurious behaviour	2.21	0.147^*^	0.126^*^	0.129^*^	0.102^*^	0.137^*^	0.129^*^	0.146^*^	1.000

*p<0.01.

### Mediating effects of emotional dysregulation traits in the association between childhood abuse and non-suicidal NSSI behaviours

Using childhood abuse as the independent variable, emotional dysregulation traits as the mediating variable and NSSI behaviour as the dependent variable, logistic regression analysis of the mediating effect model was performed using SPSS 17.0, and the results of the regression analysis were used to test whether the mediating effect was significant. The results are shown in Table 3. The mediating effect of emotional dysregulation traits in the association between childhood abuse and NSSI behaviour (a*b) was significant, and the proportion of the mediating effect to the total effect [ab/(ab + c)] was 23.23%. The direct effect of childhood abuse on NSSI behaviour mediated by emotional dysregulation traits (c) was 0.3714, which was statistically significant, indicating that emotional dysregulation traits partially mediates the association between maltreatment and NSSI behaviour. Further analysis revealed that the mediating effect of emotional dysregulation traits in the association between emotional abuse, emotional neglect, sexual abuse, physical abuse, physical neglect and NSSI behaviour was significant across all factors of childhood abuse, with the mediating effect accounting for 22.48%, 32.58%, 20.99%, 28.15%, and 24.19% of the total effect, respectively.

**Table 3:** Analysis of the intermediary effect of emotional dysregulation traits in the association between childhood abuse factors and NSSI.

Factors of childhood abuse	Emotional dysregulation traits			Emotional dysregulation traits			Proportion of intermediary effect [ab/(ab + c)] (%)
	Intermediary effect (a * b)	Lower limit	Upper limit	Direct effects (c)	Lower limit	Upper limit	
Childhood abuse	0.1124	0.0941	0.1329	0.3714^*^	0.1272	0.5878	23.23
Emotional abuse	0.1007	0.0821	0.1207	0.3927	0.1421	0.5972	22.48
Emotional neglect	0.0974	0.0625	0.1124	0.2016^*^	0.0881	0.4217	32.58
Sexual abuse	0.1021	0.0771	0.1409	0.3841^*^	0.1266	0.4079	20.99
Physical abuse	0.0944	0.0525	0.1477	0.2409^*^	0.0962	0.3271	28.15
Physical neglect	0.0832	0.0528	0.1132	0.2608^*^	0.0863	0.3156	24.19

*It means that the *p*-value of the annotated data is less than 0.01 (*p* < 0.01).

We conducted the mediation analysis separately for the FTM and MTF groups. In both groups, emotional dysregulation traits partially mediate the association between childhood abuse and NSSI behaviour, accounting for a significant proportion of the total effect. The direct effects of emotional abuse, emotional neglect, sexual abuse, physical abuse, and physical neglect on NSSI behaviour mediated by emotional dysregulation traits (c) were 0.3927, 0.2016, 0.3841, 0.2409, and 0.2608, respectively, but the direct effects of emotional abuse on NSSI behaviour were not statistically significant. This implies that emotional dysregulation traits partially mediates the association between physical abuse, sexual abuse, physical neglect and NSSI behaviour, and may fully mediate the association between emotional abuse and NSSI behaviour.

## Discussion

The results of this study showed that total childhood abuse scores were moderately associated with emotional dysregulation traits scores and weakly associated with NSSI behaviours, and that emotional dysregulation traits scores were weakly associated with NSSI behaviours, which is consistent with existing research. A cohort study showed that childhood abuse and its subtypes were associated with NSSI behaviours (Han et al., 2018), although the evidence for an association between emotional abuse and NSSI behaviours is not sufficient. Although the evidence for an association between emotional abuse and NSSI behaviour is not strong, screening for childhood abuse is an important factor in assessing NSSI risk (Han et al., 2021).

The NSSI detection rate in this study was 62.51%, which is higher than that reported in some previous studies. We posit that this could be largely due to the sample being drawn from a population in China, a country where traditional societal norms and attitudes can create substantial pressure on transgender individuals. The stigma, discrimination, and lack of social acceptance faced by transgender individuals in these traditional contexts can contribute to higher stress levels, emotional distress, and subsequent engagement in self-injurious behaviours as a coping mechanism. Regarding the assessment method, we used a single item from the DSM-5 Definitive Clinical Examination of Disorders (SCID-5-CV) to assess NSSI. This question might have a lower threshold for NSSI detection compared to other measures used in previous research, potentially contributing to the higher NSSI rate in our study. However, we believe that the significant societal factors play a more prominent role in our elevated detection rate. These findings underline the importance of understanding the social context when interpreting rates of NSSI and suggest that targeted interventions to reduce stigma and improve societal acceptance of transgender individuals in more traditional societies like China could be key in reducing NSSI rates. Future research may benefit from using multiple methods of NSSI assessment for a more comprehensive evaluation and further exploration of societal influences on these behaviours.

At the same time, patients with emotional dysregulation traits are prone to impulsive behaviours, including NSSI behaviours, and emotional instability is one of the distinctive features of the patients (Yan et al., 2019), while NSSI behaviours can quickly release them from negative reactions as a result of their poor emotional regulation (Yang et al., 2016; Cao et al., 2022; Huang, 2022). However, there is a paucity of prospective research on the two, and the relationship between emotional dysregulation traits and NSSI and the mechanisms that regulate each other are unclear and need to be determined through further research (Price et al., 2021; Zhang et al., 2021).

The impact of childhood abuse on NSSI behaviour can also be indirectly influenced by mediating factors that can affect the onset of NSSI behaviour (Espelage et al., 2021). This study suggests that childhood abuse can indirectly influence NSSI behaviour through emotional dysregulation traits (Zhang and Jiang, 2022). The present study implicitly suggests that childhood abuse may be a contributor to emotional dysregulation traits, and potentially plays a role in the pathway to Non-Suicidal Self-Injury (NSSI). For the first half of the pathway, childhood abuse increases the risk of mental illness in adulthood through cognitive and emotional pathways, and transgender people are more likely to be abused in childhood because of the social pressures they face and the low acceptance of transgender people in Chinese society (Qiang et al., 2022). In addition, emotional dysregulation traits disorder can develop when children are sexually abused as children and not dealt with appropriately. This factor explained the variance in emotional dysregulation traits symptoms. For the latter half of the pathway, emotional dysregulation traits has a very high co-occurrence with NSSI, with people with emotional dysregulation traits habitually responding to strong emotional reactions with NSSI behaviours (Rogers and Taliaferro, 2020). It has been shown that intrusions or arousals of memories associated with childhood abuse can be intensely distressing for people with BPD, that emotional dysregulation traits are a typical feature of people with BPD, and that people with BPD are prone to use poor regulation to avoid intrusive memories and thoughts. Evidence of reward mechanisms in the brain of BPD patients with NSSI behaviour also suggests that NSSI is a way for BPD patients to manage negative emotions (Wang et al., 2020).

Personality traits are developed over time and are stable, and the environment and education provided by parents and significant others are crucial to the formation of their personality. As an individual’s personality develops and matures, it is difficult for it to change. A study by Wang et al. (2021) found a high correlation between unstable personality disorder and suicidality in transgender people, especially in the context of stressful risks of discrimination and the risk of extreme behaviour. Transgender psychological problems should therefore be addressed with good coping strategies based on social awareness to reduce the likelihood of discrimination against transgender people wherever possible (Best et al., 2015). At the same time, regular psychological screening should be organised for transgender people, and if emotional dysregulation traits tendencies are detected, positive interventions can be provided to stabilise them and reduce the occurrence of NSSI behaviour, thus preventing extreme incidents (Shipherd et al., 2019).

There are some shortcomings in this study. This study did not ask detailed questions about NSSI behaviour, but only screened the NSSI population through the options in the questionnaire, and future studies need to improve the content of the questionnaire (Tantirattanakulchai and Hounnaklang, 2021). The data used in this study were collected only at the time of collection and were not followed up over time, and are cross-sectional in nature, making it difficult to reflect the impact of changes in respondents’ living conditions (Lin et al., 2021; Cao et al., 2022). Although cross-sectional studies are useful in providing insights into associations between variables at a single point in time, there are inherent limitations in using such data to make causal inferences. Cross-sectional studies cannot establish causality or directionality between variables, and the possibility of reverse causation cannot be ruled out. Moreover, cross-sectional studies do not allow for the examination of change over time, making it difficult to assess the impact of changes in respondents’ living conditions, as well as the temporal ordering of events. As noted by Maxwell et al. (2011), bias can occur in cross-sectional analyses of longitudinal mediation, particularly in cases where autoregressive models are used. Therefore, the results of this study should be interpreted with caution and considered preliminary, and future research using longitudinal designs is needed to provide a more robust understanding of the relationship between childhood abuse, emotional dysregulation, and NSSI behaviour in transgender individuals.

In-depth interviews should also be conducted with the transgender people who participated in the study. This study suggests that emotional dysregulation traits mediate the link between childhood abuse and NSSI behaviour. Although NSSI behaviour is more prevalent in transgender adolescents, childhood abuse significantly affects personality development and potential NSSI behaviour in adulthood (Calvo Piñero et al., 2021). This implies a need for proactive prevention of NSSI within the transgender community (Liu et al., 2019; Ren and Qi, 2019). Childhood experiences of abuse are irreversible, hence greater focus should be directed towards understanding the personality of transgender individuals, particularly in terms of emotional dysregulation traits, and assessing their vulnerability to societal discrimination and oppression. To reduce the stress of transgender people, the most direct method is to introduce anti-discrimination regulations. Future research will aim to address the vulnerability of transgender people to NSSI and further explore how transgender people can safeguard their psychological well-being in an environment of discrimination in order to prevent the occurrence of NSSI behaviours and extreme events.

## Conclusion

NSSI behaviours in transgender people are associated with childhood abuse and emotional dysregulation traits. Emotional dysregulation traits partially mediates the association between childhood abuse and NSSI behaviours, and therefore screening for emotional dysregulation traits in transgender people and timely intervention should be emphasized. Efforts should also be made to improve the environment for transgender people and reduce their likelihood of childhood abuse of transgender people, thereby reducing the occurrence of NSSI behaviours and extreme events in the transgender population.

## Data availability statement

The original contributions presented in the study are included in the article/supplementary material, further inquiries can be directed to the corresponding authors.

## Ethics statement

The patient in our research has signed the informed consent. This study was designed in accordance with the Declaration of Helsinki and approved by the ethics committee of Kunming University of Science and Technology. Approval number: KUST2023032128. The studies were conducted in accordance with the local legislation and institutional requirements. Written informed consent for participation was not required from these participants or the participants’ legal guardians/next of kin in accordance with the national legislation and institutional requirements.

## Author contributions

QC, YQ, NY, HG, YC, QZ, and ML: conceptualization. YC, QC, QZ, ZX, ZH, YQ, and ML: methodology. NY, HG, and ML: validation. YC, ML, and NY: formal analysis. QC, ML, and QZ: investigation. ZH, ML, and ZX: resources. ML and QC: data curation. QC, ML, ZH, ZX, YC, NY, HG, and QZ: writing—original draft. ML, YQ, HG, and NY: writing—review and editing. ML and YQ: supervision. ML, YQ, and QC: project administration. All authors contributed to the article and approved the submitted version.

## Funding

This work was supported by the Kunming University of Science and Technology 2017 Course Assessment Reform Project (KH201702).

## Conflict of interest

The authors declare that the research was conducted in the absence of any commercial or financial relationships that could be construed as a potential conflict of interest.

## Publisher’s note

All claims expressed in this article are solely those of the authors and do not necessarily represent those of their affiliated organizations, or those of the publisher, the editors and the reviewers. Any product that may be evaluated in this article, or claim that may be made by its manufacturer, is not guaranteed or endorsed by the publisher.
